# A new therapy for microcystic lymphatic malformations: the combination of intralesional laser thermolysis and percutaneous sclerotherapy

**DOI:** 10.3389/fcell.2024.1490351

**Published:** 2024-12-24

**Authors:** Zhiping Wu, Yun Zou, Ronghua Fu, Jun Cheng, Hanxiang Bai, Mengyu Huang, Hua Yuan

**Affiliations:** Department of Plastic Surgery, Jiangxi Provincial Children’s Hospital, Nanchang, China

**Keywords:** lymphatic malformations, intralesional laser thermolysis, sclerotherapy, therapy, polidocanol, pingyangmycin

## Abstract

**Background:**

Over the past few decades, percutaneous sclerotherapy has been proven to be efficacy in treating macrocystic lymphatic malformations (LMs). Unfortunately, there still remains challenging in the treatment of microcystic LMs given their size. We introduce the intralesional laser thermolysis (ILT) technique, a novel enhancement technique for the traditional percutaneous sclerotherapy in the treatment of microcystic LMs.

**Methods:**

A retrospective analysis of children with microcystic LMs treated using ILT combined with percutaneous sclerotherapy was done. All patients underwent clinically and cross-sectional imaging to assess response to treatment.

**Results:**

Between January 2020 and April 2022, 16 consecutive patients (female/male ratio: 7:9; average age, 32.6 months; range: 6 months to 16 years) with microcystic LMs received treatment with ILT combined with percutaneous sclerotherapy. A total of twenty-six sessions of combination therapy were performed, with a mean number of sessions per patient of 1.6 (±0.7), ranging from 1 to 3 sessions. The follow-up time ranged from 6 to 24 months, with a mean of 15.8 (±6.3) months. Almost all patients showed significant improvement after the combination therapy. No major complications were observed. Minor complications included circumscribed skin blisters, postoperative non-infectious fever, transient skin edema, pigmentation, and slight depressions of localized skin.

**Conclusion:**

The treatment of intralesional laser thermolysis combined with traditional sclerotherapy is considered as a safe, feasible and effective therapy in treating microcystic LMs.

## Introduction

Lymphatic malformations (LMs) are congenital lymphatic diseases often associated with somatic PIK3CA mutations (Luks et al., 2015). They occur in approximately 1 in 2,000 live births and are typically diagnosed at birth or within 2 years thereafter. LMs can manifest in any body part, with 75% affecting the head and neck region (Wu et al., 2022). According to the International Society for the Study of Vascular Abnormalities (ISSVA) classification, LMs are categorized into macrocystic (cysts > 2 cm), microcystic (cysts < 2 cm), and mixed lesions, which can be single or multifocal (McCuaig, 2017). Symptoms vary based on the size, location, and anatomy of the LMs. Small lesions may remain asymptomatic until infection or bleeding occurs, while complex lesions can lead to pain, edema, deformation, and potential airway compression. Patients with LMs in the head and neck region may experience symptoms ranging from mild swelling to severe airway obstruction, macroglossia, difficulty with oral feeding, vision loss, mandible overgrowth, aesthetic concerns, and pain (Gorostidi et al., 2022; Wiegand et al., 2023).

The primary objective in managing LMs is to address both functional impairments and aesthetic deformities. In recent years, percutaneous sclerotherapy has emerged as the preferred treatment for LMs, gradually replacing surgical resection (Caton et al., 2023a; Caton et al., 2023b; De Maria et al., 2020). However, microcystic LMs often demonstrate limited responsiveness to sclerotherapy, making surgical resection still a crucial treatment option and, in some cases, the only viable one. Despite its significance, surgical resection is not without drawbacks, as it is associated with high recurrence rates and various potential complications, including postoperative fever, infection, disfigurement, local nerve injury, and hemorrhage (Wang and Li, 2015; Bonilla-Velez et al., 2020; García-Montero et al., 2019).

Microcystic LMs typically consist of numerous small cysts (<2 cm), making them less responsive to standard sclerotherapy. In traditional percutaneous sclerotherapy, a transfusion needle is inserted into the lesion through the skin, and sclerosing agents are injected after aspirating lymph fluid (Wu et al., 2022). However, accessing the numerous very small disconnected cysts in microcystic lesions is challenging. Consequently, sclerosants struggle to enter these small channels, contributing to the poor response of microcystic LMs to percutaneous sclerotherapy. Additionally, microcystic lesions contain abundant soft tissue masses. Even if the cysts are dissolved or reduced in size, a significant amount of soft tissue remains unresponsive to sclerosing agents. Polidocanol (POL) is a non-ionic surfactant sclerosing agent known to directly damage venous endothelial cells through cellular calcium and nitric oxide signaling pathways, leading to a reduction in lesion volume. POL is also considered a safe but relatively mild sclerosant for treating LMs (Yamaki et al., 2017; De Corso et al., 2022). Pingyangmycin, derived from bleomycin A5, is an anti-tumor drug that inhibits the cell cycle by interfering with DNA synthesis (Wu et al., 2016). It induces a non-specific local inflammatory reaction in endothelial cells of the cyst wall. Pingyangmycin has also become a commonly used sclerosant for LM treatment.

In this study, we report a novel sclerotherapy technique for microcystic LMs using intralesional laser thermolysis (ILT) to destroy the wall of very small lumen to make them connected (forming artificial macrocystic LMs) and to eliminate the soft-tissue composition, then injecting sclerosants such as POL and pingyangmycin to treat the lesion.

## Materials and methods

This study retrospectively analyzed 16 patients with microcystic LMs who underwent ILT technology combined with percutaneous sclerotherapy in the Department of Plastic Surgery, Jiangxi Provincial Children’s Hospital from January 2020 to April 2022. All guardians of children were fully informed of the novelty and substitutability of this technology, and informed the possibility of adverse effects POL and Pingyangmycin, including fever, vomiting, allergies, and changes in lung function. All children undergo treatment after getting written informed consent from their parents.

This study was approved by the Institutional Review Board of Jiangxi Children’s Hospital Ethics Committee and informed consents were signed by all guardians. Meanwhile, this study was conducted in accordance with ethical guidelines of the Helsinki Declaration.

## Diagnosis

The lesion of microcystic LMs was diagnosed by the multidisciplinary team (imaging expert, interventional radiologist and surgeon of pediatry) based on physical and imaging examinations. Diagnostic criteria were based on physical, ultrasound and magnetic resonance imaging (MRI) examinations, such as clinical symptoms, vesicles on skin, solid cystic lump with lymph or hemorrhagic fluid, and fluid signals on MRI. The extensive and structure of lesion was assessed by the imaging studies. There was no preoperative biopsy in all patients.

### Technique

**FIGURE 1:**
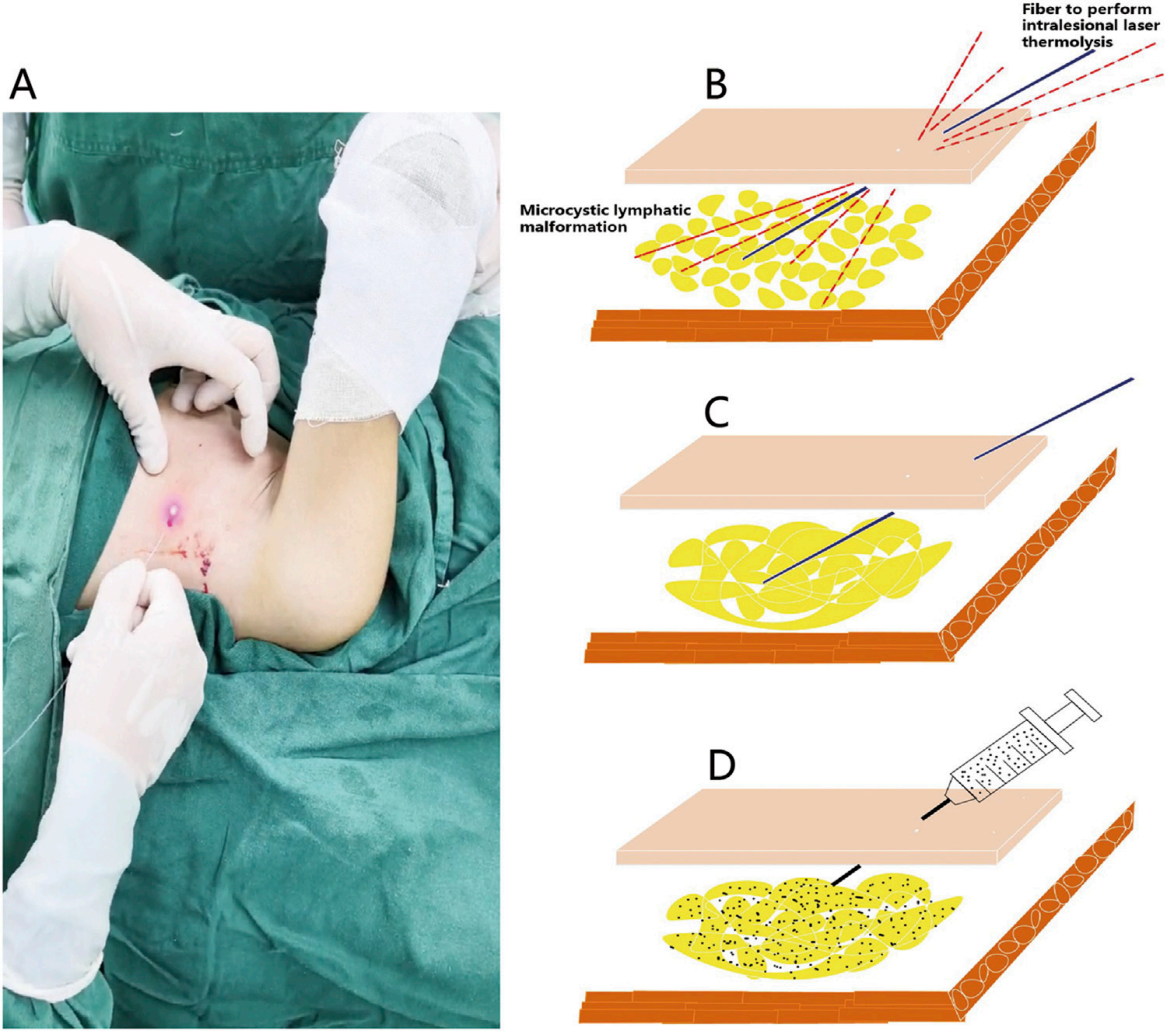
Operative technique of the intralesional laser thermolysis combined with percutaneous sclerotherapy. **(A)** Photograph of the operation of intralesional laser thermolysis. **(B)** After topographic marking and tumescent infiltration, the operation of intralesional laser thermolysis was performed. **(C)** The wall of lymphatic malformations was destroyed after intralesional laser thermolysis. **(D)** Showing the diffusion of the sclerosant of pingyangmycin and polidocanol in the treatment area.

**FIGURE 2:**
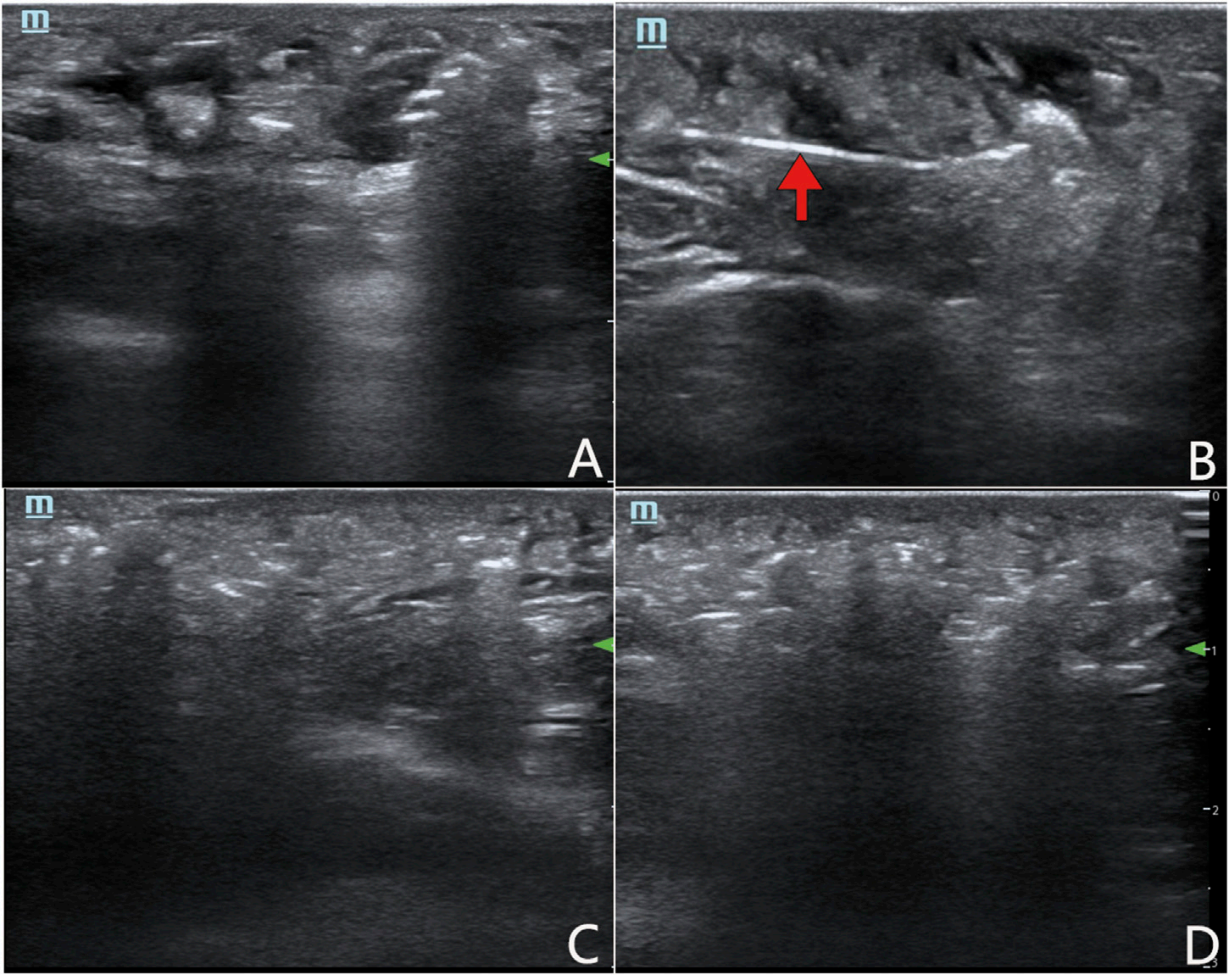
Ultrasound examinations to confirm the condition of damaged small lumens and soft tissue in microcystic LMs. **(A)** The area of microcystic LMs before the operation of intralesional laser thermolysis. **(B)** The fiber to perform intralesional laser thermolysis (arrow). **(C)** The area of microcystic LMs after intralesional laser thermolysis. **(D)** Showing the diffusion of the sclerosants in the treatment area.

The LASEmaR 1,000 is a diode laser device manufactured by EUFOTON S.R.L. (Italy), which operates at a wavelength of 980 nm. This wavelength is effectively absorbed by targeted tissues, generating thermal energy that causes coagulation and vaporization, which disrupts the lesion. The device is compact and portable, making it suitable for use in both hospital and outpatient settings, offering flexibility for practitioners to perform procedures across various clinical environments. It uses specialized, single-use fiber-optic applicators, which can be directly inserted into the lesion. The fiber’s diameter and length vary depending on the size of the lesion and treatment area. Additionally, the device allows for customizable settings, including power output, pulse duration, and spot size, to optimize treatment outcomes and minimize complications. In our study, we employed the LASEmaR 1,000 with a 600 μm fiber for ILT of microcystic LMs. The laser was set to operate in continuous mode, with a power range of 6–10 W, to target the lesions effectively. Prior to the procedure, ultrasonologists marked the lesion’s location and extent with permanent markers. All surgeries were conducted in a dedicated operating room under general anesthesia to ensure optimal conditions for pediatric patients. To achieve the best exposure to the treatment area and facilitate surgical evaluation, the patient’s preoperative position was determined by the operators. Similar to laser lipolysis or liposuction procedures, we administered tumescent solution local anesthesia in the affected areas before the operation (Wolfenson et al., 2015; Mordon and Plot, 2009). This tumescent solution consisted of lidocaine, epinephrine, and saline (2% lidocaine 5 mL + 1:1,000 epinephrine 1 mL + saline 1,000 mL). To maximize the vasoconstrictive effect, the procedure was performed at least 10 minutes after injection (Klein and Jeske, 2016). Next, the laser fiber was inserted into the region through perforations in normal skin near the lesion site. We created multiple perforations and laser tunnels in various directions to ensure complete coverage of the lesion (Figure 1). In cases of significant resistance, the energy output was appropriately increased to ensure the destruction of the dense walls of small cysts and soft tissue. Both patients and the surgeon wore protective eyewear to prevent laser wavelength-related injuries. We used pinch tests, in which the clinician uses their thumb and index finger to pinch the skin and underlying soft tissue in the treated lesion, and ultrasound examinations to confirm the condition of damaged small lumens and soft tissue in the area (Figure 2). The endpoint of the procedure was defined as the pinch test and ultrasound indicating that the lesion had been destroyed and the treated area had become flat and smooth in patients with cutaneous symptoms. To prevent unintended blistering on the treated skin, we applied ice gauze to reduce the temperature of the lesion area.

Following the ILT, we performed percutaneous injections using a single transfusion needle and a double or multiple syringe system under ultrasound guidance. To ensure the thorough distribution of the sclerosant within the lesion, we administered multiple percutaneous injections from various directions surrounding the primary lesion. To prevent the overflow of sclerosing agents from the small perforations after completing the procedure, we applied local compressive dressings. The total recommended dose of pingyangmycin should not exceed 40 mg (typically 0.5 mg/kg) (Zheng et al., 2009), while the maximum allowable dose of POL is 2 mg/kg (Rabe et al., 2014).

### Postoperative care and follow up

Antibiotics were not administered in this case series. Instead, routine cold compress was applied during the first 6 hours post-procedure to reduce excessive local swelling and delay the absorption of sclerosants by blood vessels. Guardians were instructed to monitor for post-operative complications, such as edema, ecchymosis, pigmentation, or re-enlargement. Patients returned for a 1-month follow-up assessment after discharge, and all underwent MRI examinations 6 months after the procedure. The follow-up period ranged from 6 to 24 months, with a mean duration of 15.8 (±6.3) months.

To evaluate treatment effectiveness, two independent imaging experts assessed imaging examinations. Postoperative MRI findings were categorized into three response levels: sub-complete response (lesion reduction >80%), partial response (25%–80%), and no response (<25%). The initial response to sclerotherapy was determined based on the 6-month follow-up MRI, and additional sclerotherapy sessions were performed if necessary. The final treatment outcome was evaluated by a multidisciplinary team comprising pediatric interventional radiologists, sonography experts, and pediatric surgeons.

## Results

Between January 2020 and April 2022, a total of 16 patients with microcystic LMs were treated with a combination of ILT and percutaneous sclerotherapy. The characteristics of the patient cohort are summarized in Table 1. The mean age at the time of initial treatment was 32.6 months (range: 6 months to 16 years). A total of 26 combination therapy sessions were performed across all patients, with a mean of 1.6 (±0.7) sessions per patient, ranging from 1 to 3 sessions. The follow-up period ranged from 6 to 24 months, with a mean duration of 15.8 (±6.3) months.

**TABLE 1:** Summary of patients with microcystic lymphatic malformations treated with the intralesional laser thermolysis technique.

Patient Number	Sex	Age (years)	Site	Symptoms	Number of sessions	Subsequent surgical treatment	Response	Transient mild complications (fever or vomiting)	Follow-up (months)
1	Female	16	Abdominal wall	Soft tissue mass, oozing	3	No	Partial response	No	12
2	Female	2	Right buttock	Soft tissue mass	1	No	Sub-complete response	Fever (>39.0°C) during the first day	6
3	Male	0.5	Right thigh	Pain, oozing	2	Yes	Sub-complete response	No	18
4	Male	3	Left forearm	Soft tissue mass, pain	3	No	Sub-complete response	No	18
5	Female	1	Left buttock	Soft tissue mass	1	No	Partial response	Vomiting	12
6	Male	0.5	Left upper arm	Soft tissue mass	1	No	Sub-complete response	No	24
7	Male	1.5	Right buttock	Soft tissue mass	2	No	Sub-complete response	No	18
8	Female	9	Right knee	Pain, functional impairment	1	No	Sub-complete response	No	18
9	Male	5	Abdominal wall	Soft tissue mass, oozing	2	Yes	Sub-complete response	No	24
10	Male	3	Left thigh	Pain, functional impairment	1	No	Partial response	Fever (>39.0°C) during the first day	6
11	Female	2	Left knee	Soft tissue mass	2	No	Sub-complete response	No	12
12	Female	2	Left lower leg	Soft tissue mass	1	No	Partial response	Vomiting	24
13	Male	0.5	Right forearm	Soft tissue mass	2	Yes	Sub-complete response	No	18
14	Male	2	Right knee	Soft tissue mass, pain	2	No	Sub-complete response	No	24
15	Female	3	Left thigh	Pain	1	No	Partial response	Fever (>39.0°C) during the first day	6
16	Male	2	Right thigh	Soft tissue mass	1	No	Sub-complete response	No	12

Post-treatment MRI evaluations indicated favorable outcomes, including a reduction in lesion size, for most patients (Table 1). Although three patients underwent surgical resection following combination therapy, it was not possible to isolate the effects of the combination therapy from those of the surgery. Outcomes for the remaining 13 patients generally depended on lesion size and the number of treatment sessions. Specifically, 11 patients (68.8%) exhibited a sub-complete response, defined as a reduction in lesion size of greater than 80%. Five patients (31.2%) achieved a partial response, with a 25%–80% reduction in lesion size. No patients experienced minimal or no change in lesion size.

**FIGURE 3:**
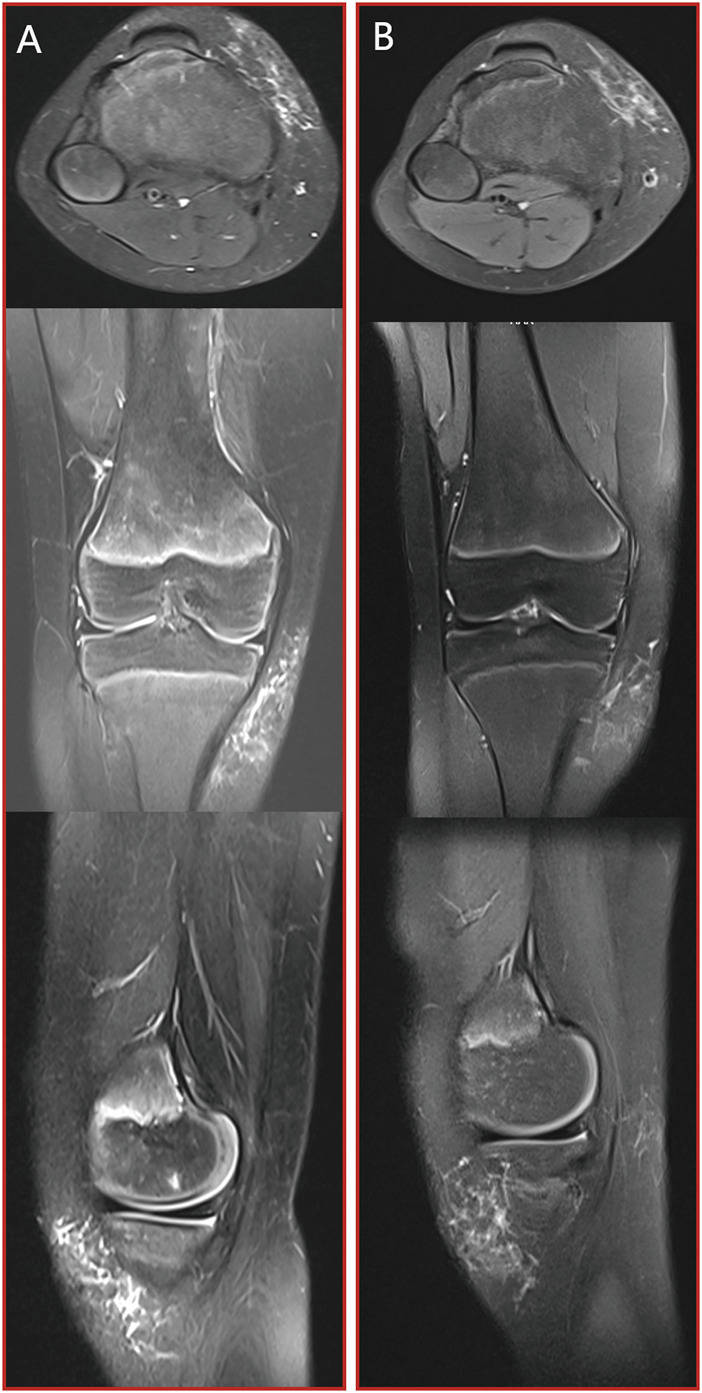
Case 1: patient with microcystic LMs of the right knee which achieved a substantial and noteworthy improvement after a single session of combination therapy. **(A)** pre-treatment MRI stud; **(B)** post-treatment MRI study. From top to bottom: axial T2 fat saturated image, coronal T2 fat saturated images and sagittal T2 fat saturated images.

To better illustrate the clinical outcomes observed in this study, the following case examples highlight the treatment response and notable improvements following combination therapy:

Case 1 A 9-year-old girl sought medical attention due to a persistent right knee pain and functional impairment, a condition she had been experiencing for the past 4 years. Upon further examination through MRI imaging, the findings revealed the presence of a microcystic LMs, characterized by the typical ‘sunburst’ microchannels observable on T2 MRI scans (as shown in Figure 3A). To address this condition, the patient underwent a single session of combination therapy. Remarkably, following this treatment, there was a substantial and noteworthy improvement in the patient’s clinical condition, which is clearly demonstrated in Figure 3B.

**FIGURE 4:**
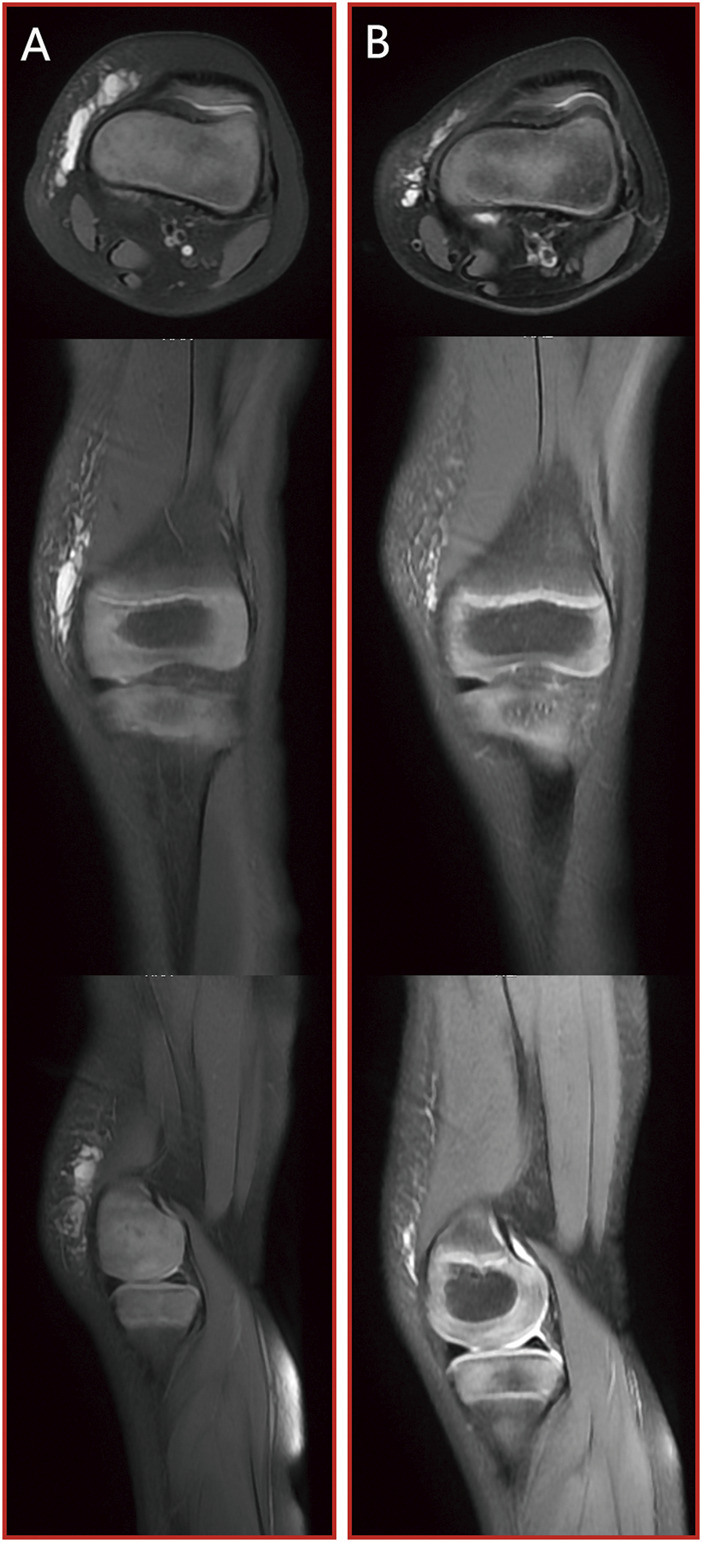
Case 2: patient with microcystic LMs of the left distal medial thigh which achieved a notable reduction in the size of the intraconal cystic component after two sessions of combination therapy. **(A)** pre-treatment MRI stud; **(B)** post-treatment MRI study. From top to bottom: axial T2 fat saturated image, coronal T2 fat saturated images and sagittal T2 fat saturated images.

Case 2 A 2-year-old girl was brought to our attention due to the presence of a soft tissue mass located on left knee. An MRI examination revealed a lesion characterized by prominent T2 fluid signal intensity, as depicted in Figure 4A. Following two sessions of combination treatment, a subsequent MRI with T2 imaging demonstrated a notable reduction in the size of the intraconal cystic component, as seen in Figure 4B.

**FIGURE 5:**
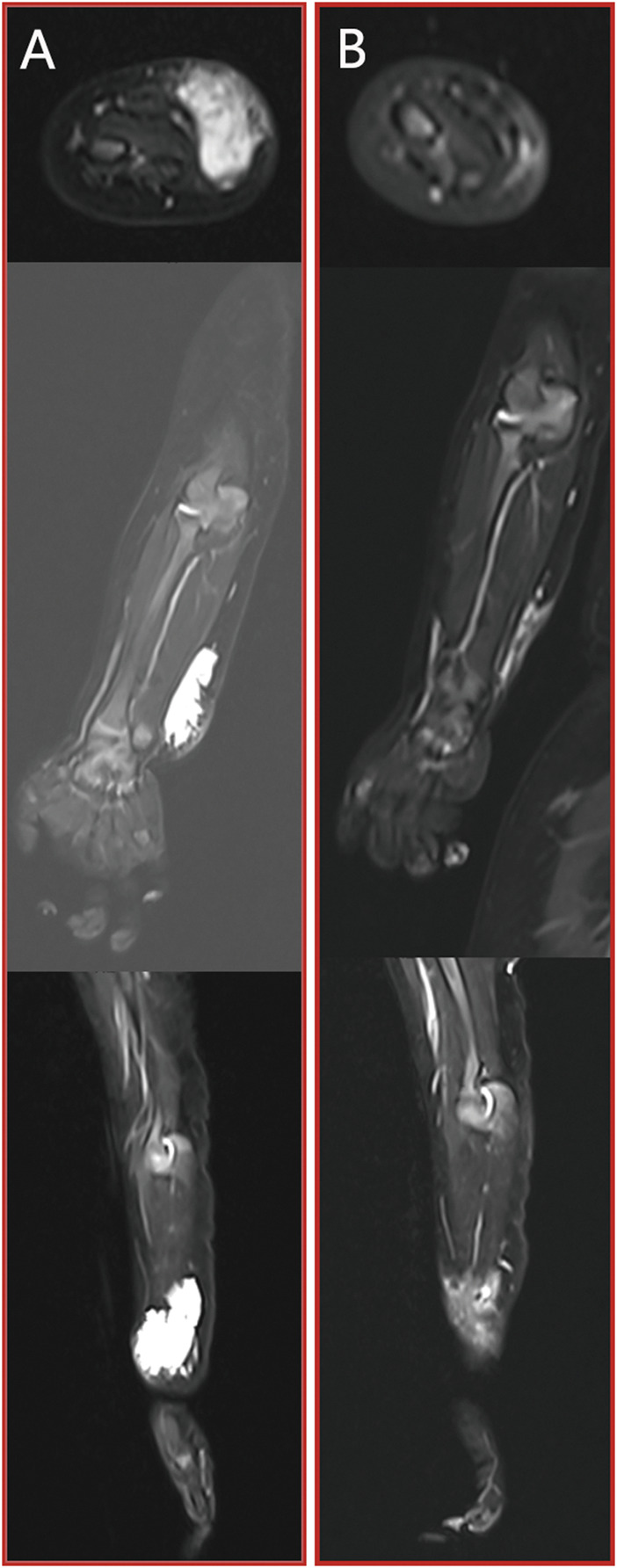
Case 3: patient with microcystic LMs of the right upper medial wrist joint which achieved a marked reduction in the size of the lesion after two sessions of combination treatment and surgical resection. **(A)** pre-treatment MRI stud; **(B)** post-treatment MRI study. From top to bottom: axial T2 fat saturated image, coronal T2 fat saturated images and sagittal T2 fat saturated images.

Case 3 At birth, a young child was diagnosed with an extensive microcystic LMs affecting the soft tissue mass of the right forearm. This condition was evident on MRI scans by the presence of a distinct bright T2 fluid signal intensity, as illustrated in Figure 5A. After undergoing two sessions of combination treatment, surgical resection was performed. Following the final treatment, a subsequent MRI with T2 imaging revealed a marked reduction in the size of the lesion, accompanied by the presence of residual intraconal low signal intensity fibrous tissue, as demonstrated in Figure 5B.

**FIGURE 6:**
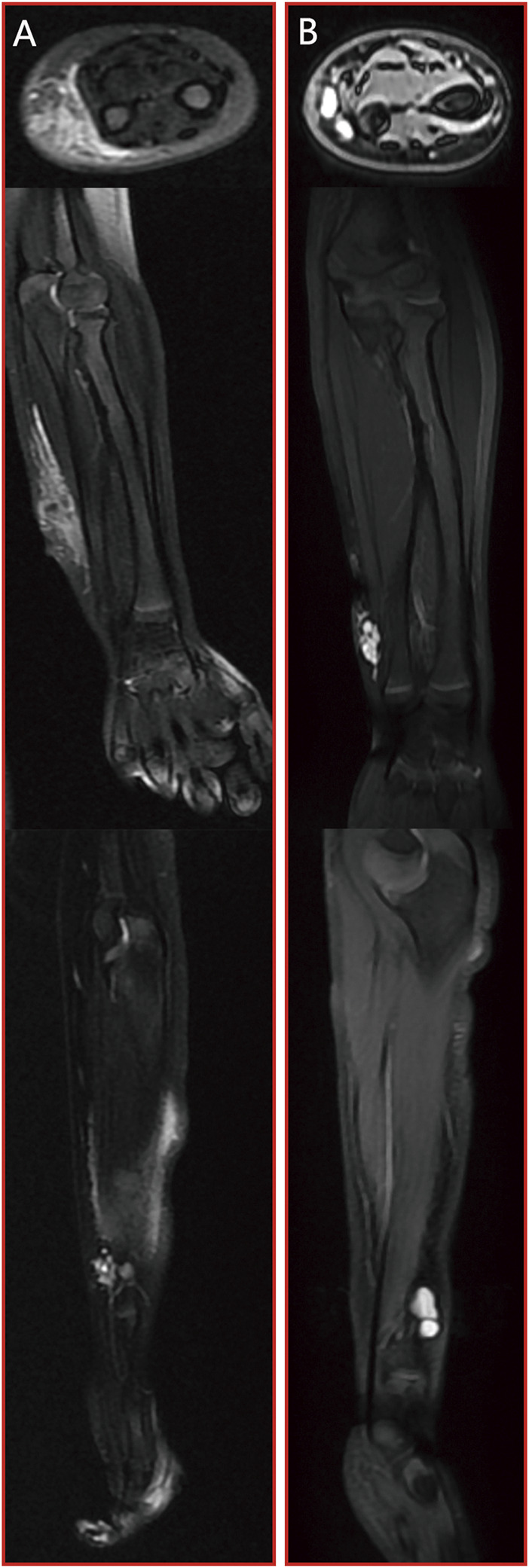
Case 4: patient with microcystic LMs of left forearm which achieved a significant and remarkable improvement after three sessions of combination treatment. **(A)** pre-treatment MRI stud; **(B)** post-treatment MRI study. From top to bottom: axial T2 fat saturated image, coronal T2 fat saturated images and sagittal T2 fat saturated images.

Case 4 We were alerted to the case of a 3-year-old boy who presented with a soft tissue mass and pain in his left forearm. Upon conducting MRI scans, it became evident that the lesion displayed a distinct and prominent bright T2 fluid signal intensity, as visualized in Figure 6A. Subsequently, the patient underwent a series of three combination treatment sessions. Remarkably, following the completion of the final treatment session, a significant and remarkable improvement in the patient’s clinical condition was observed. This substantial progress is clearly illustrated in Figure 6B.

## Complications

There was no major complication in these case series, such as distal embolization, local tissue necrosis, or thinning of the skin. Most patients experienced transient skin edema, pigmentation, mild localized depression and/or irregularity, and local skin blisters. The pigmentation in the treatment area gradually subsided, but lasted for at least a few months or more with no intervention. Other temporary mild complications included severe non-infectious fever (>39.0°C) in 3 patients within 24 h after operation, vomiting in 2 patients that alleviated within 24 h after non-specific intravenous infusion (Table 1). During the follow-up period, four patients with a wide range of lesion showed excessive skin in the treatment area.

## Discussion

The term “microcystic LMs” was introduced by Fox and Fox in 1878, with Morris describing lymphangioma circumscriptum in 1889 (Fox and Fox, 1879; Whimster, 1976). The current and appropriate terminology for lymphatic malformations found in the skin, subcutaneous tissue, and sometimes muscle areas is “microcystic LMs” (Peachey et al., 1970). These malformations typically present as a localized, painless mass. In some cases, vesicles resembling frog eggs or warts, containing clear fluid or blood, may appear on the skin or mucous membranes. These vesicles are believed to originate from deeper, contractile lymphatic sources that deviate from the normal lymphatic system in the surrounding tissue (Whimster, 1976). Historically, radical surgery has been the preferred treatment for microcystic LMs, despite a recurrence rate of up to 17%, which depends on the lesion’s extent (Browse et al., 1986). Surgical excision involves penetrating through the entire skin layer, subcutaneous tissue, and fascia to remove abnormal lymphatic tissues (Browse et al., 1986). However, this approach presents challenges, particularly for extensive infiltration lesions.

Sclerotherapy alone has also been considered a treatment option for microcystic LMs, utilizing hypertonic saline, doxycycline, picibanil, and bleomycin (Molitch et al., 1995; Lam and Yuen, 2019; Golinelli et al., 2015; Khurana et al., 2018). However, the efficacy of percutaneous sclerotherapy for these lesions is generally lower, and the incidence of complications is higher. This can be attributed, in part, to the traditional percutaneous sclerotherapy technique, which involves entering the cyst and injecting sclerosing agents. This method yields limited success in treating microcystic LMs, primarily due to the presence of small cysts where sclerosing agents struggle to diffuse effectively within the lesion (Wang et al., 2022). Another possible factor contributing to this limited efficacy is the excess soft tissue found in these lesions, which may not respond adequately to sclerosant treatment.

Shapshay was the first to report on intralesional laser therapy for vascular abnormalities (Shapshay et al., 1987). Since then, intralesional laser treatment has been employed in various vascular abnormalities, either as a standalone treatment or in combination with other supportive methods (Burstein et al., 2000; Wada et al., 2020; Sun et al., 2022). However, to date, there have been no reports on the use of intralesional laser therapy for microcystic LMs. Drawing upon our prior successful experiences in treating vascular tumor, such as kaposiform hemangioendothelioma (KHE) and tufted angioma (TA), as well as vascular malformations, we adopted ILT in conjunction with percutaneous sclerotherapy to address pediatric patients with microcystic LMs. Considering the pathological characteristics of microcystic LMs, which typically comprise numerous small cysts and an abundance of soft tissue components, intralesional laser therapy serves as a promising approach. Through the use of optical fibers, laser energy can be directly delivered to the depths of the lesion, effectively disrupting the integrity of small cysts and enhancing the contact between sclerosing agents and cyst walls (Wang et al., 2022). Simultaneously, this method can eliminate the excess soft tissue within the lesion. Additionally, the tumescent solution exerts a vasoconstrictive effect, delaying the absorption of sclerosing agents through neurovascular bundles and thereby prolonging the retention time of sclerosants within the lesion.

The administration of POL foam and pingyangmycin into subcutaneous soft tissue can result in varying degrees of damage to normal tissue, but it may not be potent enough to induce necrosis of the skin (Chaudry et al., 2014; Jin et al., 2018). The skin overlaying the treatment area, along with its associated adnexal structures, receives nourishment from the reticular structure of neurovascular bundles that remain *in situ*. In cases of microcystic LMs treated with traditional percutaneous sclerotherapy, there are often very small cysts that are difficult to access, even with ultrasound guidance. Consequently, it is quite common for sclerosing agents to extravasate into the normal tissues surrounding the lesion during standard percutaneous sclerotherapy for microcystic LMs. However, in the case of microcystic LMs, there have been few reports of skin necrosis following intralesional administration of POL foam and pingyangmycin within the lesion. Similarly, no instances of skin necrosis were observed in this research.

Injecting sclerosant after ILT allows for a more even and deeper delivery of the sclerosing agent into the numerous small cysts that become connected after the operation. A wide distribution of the sclerosant, with relatively low sclerosant concentration, can help minimize adverse effects, such as subcutaneous fibrosis. In our cases, the skin in the operated region regained its pinchable quality during the recovery period. The removal of excess soft tissue from the treatment area also contributes to the restoration of appearance. Built upon these principles, the combined technique offers a broader treatment area and more effective management of a wide range of microcystic LMs lesions compared to traditional percutaneous sclerotherapy. Several percutaneous techniques have been reported for the treatment of microcystic LMs, including intralesional injection of sclerosant and lymphographic-like sclerotherapy with doxycycline, bleomycin, or OK-432 (Chaudry et al., 2014; Burrows et al., 2008; Da Ros et al., 2018). However, multiple treatments are often required, especially for extensive lesions that contain extremely small lumens. As the cumulative dose of sclerosing agents increases, the risk of sclerosant-related complications also rises (Chaudry et al., 2014).

Intralesional laser thermolysis within the lesion is, to some extent, a technique that lacks direct visual guidance, which raises the potential risk of inadvertently damaging surrounding tissues. As a precautionary measure, ultrasound navigation should be employed during this procedure to mitigate the risk of nerve damage (Wada et al., 2020). For instance, Burstein’s report documented two cases of facial nerve injury among one hundred patients undergoing intralesional laser treatment in facial areas (Burstein et al., 2000). Fortunately, in our study, we did not observe any instances of nerve damage. Another complication that requires careful consideration is cutaneous burns. To address this concern, we adhered to recommended guidelines, ensuring that the laser fibers were positioned 5–10 mm below the lesion’s surface. Additionally, we employed ice cooling to reduce skin temperature and minimize the risk of thermal damage. Importantly, none of our cases experienced iatrogenic ulcers, perforation, or hemorrhage. During and after the treatment, there is a possibility of potassium release from intracellular to extracellular environments due to hemolysis, potentially influenced by ILT and certain cytokines or inflammatory factors. Therefore, it is advisable to conduct an electrocardiogram, urine test, and blood test (including a complete blood cell count, potassium concentration assessment, disseminated intravascular coagulation evaluation, and renal function assessment) immediately after treatment and the following morning.

This study has several limitations that should be acknowledged. Firstly, it is important to note that this study is a retrospective review of a case series, which may inherently have certain biases and limitations associated with this study design. Secondly, a standardized criteria or response evaluation tool was not employed, making it challenging to directly compare our results with those of other reports in the field. Therefore, there is a need for prospective studies that include a comparison of the combination of intralesional laser thermolysis and percutaneous sclerotherapy with other management approaches. Such studies are anticipated to provide more robust and convincing results, allowing for a more comprehensive evaluation of the effectiveness of the treatment strategy.

## Conclusion

The treatment of microcystic LMs remains challenging and requires a new treatment technique. We report a new technique for treating these lesions. The treatment of intralesional laser thermolysis combined with traditional sclerotherapy is a safe, feasible and effective therapy in treating microcystic LMs. And a prospective study is necessary to compare traditional percutaneous sclerotherapy with this new technique.

## Data Availability

The raw data supporting the conclusions of this article will be made available by the authors, without undue reservation.
